# MicroRNA-655 attenuates the malignant biological behaviours of retinoblastoma cells by directly targeting PAX6 and suppressing the ERK and p38 MAPK signalling pathways

**DOI:** 10.3892/or.2022.8385

**Published:** 2022-08-08

**Authors:** Min Zhang, Qiongxia Li, Yingzhe Pan, Hui Wang, Gang Liu, Hui Yin

Oncol Rep 39: 2040–2050, 2018; DOI: 10.3892/or.2018.6264

Following the publication of this paper, it was drawn to the Editors' attention by a concerned reader that data shown in various of the panels portraying the results from flow cytometric experiments in Fig. 2D, 5D and 6D were strikingly similar to data appearing in different form in other articles by different authors. Owing to the fact that the contentious data in the above article had already been published elsewhere, or were already under consideration for publication, prior to its submission to *Oncology Reports*, the Editor has decided that this paper should be retracted from the Journal. The authors were asked for an explanation to account for these concerns, but the Editorial Office did not receive a reply. The Editor apologizes to the readership for any inconvenience caused.

